# Modulation Steering Motion by Quantitative Electrical Stimulation in Pigeon Robots

**DOI:** 10.3390/mi15050595

**Published:** 2024-04-29

**Authors:** Mingxuan Bi, Huimin Zhang, Yaohong Ma, Hao Wang, Wenbo Wang, Yuan Shi, Wenlong Sheng, Qiushun Li, Guangheng Gao, Lei Cai

**Affiliations:** 1 Biology Institute, Qilu University of Technology (Shandong Academy of Sciences), Jinan 250100, China; 10431211245@stu.qlu.edu.cn (M.B.); 10431211038@stu.qlu.edu.cn (H.Z.); myh13322@163.com (Y.M.); shengwenlong1121@163.com (W.S.); liqs@sdas.org (Q.L.); hgg5738@163.com (G.G.); 2 College of Mechanical and Electrical Engineering, Nanjing University of Aeronautics and Astronautics, Nanjing 211100, China; haowang@nuaa.edu.cn (H.W.); wwb523@nuaa.edu.cn (W.W.); 3 School of Life Sciences, Qilu Normal University, Jinan 250200, China; cornelius7991@outlook.com

**Keywords:** pigeon, animal robots, electrical microstimulation, gradient voltage, steering control

## Abstract

The pigeon robot has attracted significant attention in the field of animal robotics thanks to its outstanding mobility and adaptive capability in complex environments. However, research on pigeon robots is currently facing bottlenecks, and achieving fine control over the motion behavior of pigeon robots through brain–machine interfaces remains challenging. Here, we systematically quantify the relationship between electrical stimulation and stimulus-induced motion behaviors, and provide an analytical method to demonstrate the effectiveness of pigeon robots based on electrical stimulation. In this study, we investigated the influence of gradient voltage intensity (1.2–3.0 V) on the indoor steering motion control of pigeon robots. Additionally, we discussed the response time of electrical stimulation and the effective period of the brain–machine interface. The results indicate that pigeon robots typically exhibit noticeable behavioral responses at a 2.0 V voltage stimulus. Increasing the stimulation intensity significantly controls the steering angle and turning radius (*p* < 0.05), enabling precise control of pigeon robot steering motion through stimulation intensity regulation. When the threshold voltage is reached, the average response time of a pigeon robot to the electrical stimulation is 220 ms. This study quantifies the role of each stimulation parameter in controlling pigeon robot steering behavior, providing valuable reference information for the precise steering control of pigeon robots. Based on these findings, we offer a solution for achieving precise control of pigeon robot steering motion and contribute to solving the problem of encoding complex trajectory motion in pigeon robots.

## 1. Introduction

In the field of neuroscience and bioengineering, researchers have been pursuing a challenging goal: to control the behavior of organisms by manipulating their nervous systems [1]. The development of neural stimulation can be traced back more than 150 years. Since the discovery of bioelectricity, the understanding and exploration of its impact on animal brain function have progressed [2,3]. With the flourishing development of neural implantation techniques, researchers have successively explored the field of neural stimulation through methods such as recording electrodes [4], transcranial drug delivery [5], and brain–computer interfaces [6]. Brain–computer interface (BCI) technology has provided a reliable solution for the regulation of biological motor behaviors, enabling an induction of animal motor behaviors [7,8]. Based on this principle, extensive research on neural implantable devices has advanced the development of animal robots [9,10]. Animals such as insects [11,12], geckos [13], rat [14], and pigeons [15] are widely used. Small animal robots exhibit outstanding adaptability to diverse application scenarios, can achieve effectively autonomous obstacle avoidance in complex environments, and can mitigate against external interference, making them a popular choice for disaster relief and topographic surveys [16,17,18,19,20]. Animal robots have become a highly anticipated frontier field, and the research on pigeon robots is also in full swing [15,21,22,23,24]. At present, the research on pigeon robots is facing a bottleneck, and it is still difficult to realize the fine control of the flight action of it through the brain–computer interface. Most research on pigeon robots is currently in the qualitative stage, and further work is needed to quantitatively analyze the correlation between electrical stimulation and behavioral actions [24]. To delve deeper into the research, it is necessary to systematically quantify the relationship between electrical stimulation and motor behavior, so as to achieve an accurate coding of pigeon motor behavior.

Further development of animal robots is closely linked to advancements in the field of neuroscience. Decoding the regulatory mechanism of the animal brain on motor behavior is a firm cornerstone for the future development of animal robots. As a common animal model in neuroscience research, the pigeon has been used to preliminarily establish the histological and anatomical structure of its central nervous system [25,26]. Previous research indicates that achieving motion control in animal robots through electrical stimulation methods relies on three key factors: stimulation sites, stimulation pattern, and stimulation parameters [27]. There are significant differences in the responses to the electrical stimulation of specific brain nuclei in different regions. The accurate positioning of the stimulation site in the brain will determine the sensitivity of animal robot behavior response. In the research on pigeons, the posterior amygdala (PoA) and the dorsalis intermedius ventralis anterior (DIVA) of the somatic nociceptive area after stimulating the fear sensing area successfully controlled the movement behavior of pigeons, such as taking off or turning [21,28]. However, it is important to note that the movement mechanism may be influenced by fear and pain. Prolonged stimulation may lead to cognitive bias in pigeons due to potential conflicts between the stimulation signal and the information received by the animal’s own sensory organs, thus affecting the effectiveness of movement behavior control [15]. Located in the midbrain motor area, the formatio reticularis medialis mesencephali (FRM) of the midbrain can induce ipsilaterally circling in pigeons. Recently, FRM has been selected as the main regulatory nucleus in the mainstream regulation scheme of mechanical pigeons [23,24]. Furthermore, the establishment of different stimulation parameters also affects the differences in motor responses. It has been demonstrated that precise control of the turning angle of a rat robot can be achieved by quantitatively adjusting the electrical stimulation parameters of the ventral posteromedial nucleus (VPM) [29]. Similarly, under outdoor conditions, the size of the turning angle during the flight of a pigeon robot varies with different stimulation parameters [23]. However, up to now, the research on the movement-related nuclei in the pigeon brain and the motor control loop of the nervous system is not enough. It is still a challenge to accurately control the flight motion of a pigeon robot. In order to solve this problem, it is necessary to quantify the relationship between electrical stimulation and motor response.

In this study, we used a BCI radio stimulation system to induce the movement of pigeons. Subsequently, we applied gradient voltage intensity electrical stimulation to elicit different behaviors in pigeons. We then quantitatively analyzed the influence of varying voltage intensities on pigeons’ steering movement behavior. Additionally, we discussed the response time of the electrical stimulation and the validity period of the BCI. The objective of this study is to present an analytical method to demonstrate the effectiveness of a pigeon robot controlled by electrical stimulation and to propose a solution for accurately controlling the motion behavior of the pigeon robot.

## 2. Materials and Methods

### 2.1. Experimental Subjects

For this study, N = 6 adult pigeons (*Columba livia*) of undetermined sex were obtained from the Biology Institute of Shandong Academy of Sciences. The pigeons ranged in age from 1 to 4 years, weighing 470 ± 80 g.

Animal care methods: The pigeons were raised in a collective pigeon loft (approximately 21.0 m^2^ in area), which was maintained in a semi-enclosed state and provided environmental conditions of 10–25 °C. Pigeons were fed with a blend of grains primarily consisting of corn, soybeans, wheat, and sorghum. The pigeon loft was equipped with a fully automated feeding and watering system to ensure an adequate supply of food and water. Animals were checked daily for signs of activity and general health and fed three times per day. Prior to surgery, the birds fasted for 24 h and drank freely during this period. During the next training and testing, they were in a naturally fed state.

Euthanasia protocols and standards for experimental animals: upon completion of the behavioral experiments, histological brain sections were performed on all subjects to validate the accuracy of electrode implantation sites.

After all experiments were completed, the subjects were injected with an overdose of pentobarbital sodium solution (1.5%), and the brains were fixed by perfusion of the physiological salt solution followed by 4% formaldehyde. All efforts were made to minimize animal suffering. In the present experiment, all pigeons (N = 6) were euthanized at the conclusion of the study. There were no instances of irreversible health conditions or severe distress among the experimental animals, obviating the need for euthanasia prior to or during the experiment.

All procedures in this research, from surgical implantation to euthanasia, were conducted in accordance with the Guide of Laboratory Animal Management Ordinance of China. All data were collected under The Biology Institute, Shandong Academy of Sciences IACUC approval SWS20220628.

### 2.2. The Electrode Adapter

In order to achieve wireless control of pigeons to induce steering and flight movements, an electrode adapter that can be permanently fixed to the pigeon’s skull was designed [30]. The adapter device consists of a customized single-layer printed circuit board (PCB), fixed screws, female header 1 × 9P-4.5 (single female header, spacing 1.27 mm, 9 holes, height 4.5 mm), and pin header S1 × 9P-6/3 (single pin header, 9 pin, height 6.0 mm, height of fitting end 3.0 mm). The PCB can be combined with the stimulation electrode to make an 8-channel stimulation system, which is suitable for both matching with the output port of the wireless stimulation system and facilitating the fixation of the electrode inside the brain. The electrode adapter is simple in structure and easy to assemble, enabling independent stimulation of eight regions of the pigeon’s brain.

### 2.3. Electrode Adapter Installation Scheme

**Figure 1:**
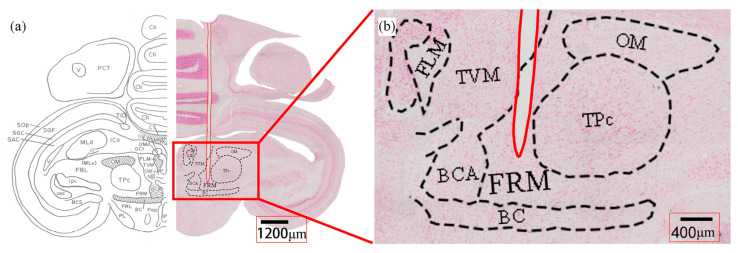
Pigeon brain sections (neutral red staining). (**a**) Histology of pigeon brain to confirm electrode insertion coordinates. (**b**) A clear needle track can be found in red solid line, which resulted from long-term implantation of the electrode. Formatio reticularis medialis mesencephali (FRM), Fasciculus longitudinalis medialis (FLM), Tractus vestibulo-mesencephalicus (TVM), Brachium conjunctivum ascendens (BCA), Brachium conjunctivum (BC), Nucleus nervi oculomotorii (OM), Nucleus tegmenti pedunculo-ponticus, pars compacta (TPc).

**Figure 2:**
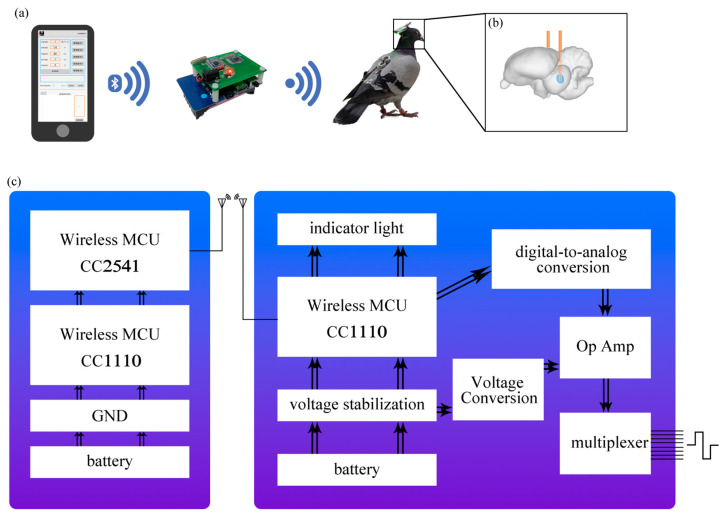
Wireless stimulation system schematic. (**a**) Schematic diagram of wireless electrical stimulation experiment. (**b**) Schematic diagram of electrode implantation FRM. (**c**) Block diagram of the stimulation system.

Anesthesia was induced using a 1.5% sodium pentobarbital and 0.8% NaCl mixture, administered intramuscularly at a dosage of 2 mL/kg of body weight. Once anesthetized, a mixed solution of 0.4 mL of 2% lidocaine hydrochloride and 0.1 mL of 5% adrenaline hydrochloride was injected subcutaneously in the surgical area for local anesthesia. Subsequently, the pigeons were placed in a brain stereotaxic apparatus (Type 68027, RWD Life Science, Shenzhen China) after feather removal and cranial skin exposure. A minimally invasive craniectomy was performed at specific locations based on the target structure, using the bregma as the reference point for pigeon brain localization. The FRM area was identified as the stimulation target according to the pigeon brain atlas. Burr holes were drilled above the target brain area, and screws were implanted on the skull surface to serve as reference poles. Nickel chromium alloy electrodes were then implanted into the FRM on both sides, ensuring coverage of core and sub areas (Figure 1a,b and Figure 2b). Electrodes were incrementally inserted using manual propellers, with a negative square-wave pulse delivered upon reaching the desired depth. After fixation with α-cyanoacrylate quick medical adhesive and glass-ionomer cement, the exposed skull was covered with a thin layer of dental acrylic. Then the electrode adapter was secured with dental acrylic, and the free ends of the electrodes were soldered to the corresponding channels of the adaptor. Post-surgery, the surgical incision was disinfected with Lincomycin Hydrochloride and Lidocaine Hydrochloride Gel, and pigeons received an intramuscular injection of 0.3 mL gentamicin sulfate to prevent infection. After one week convalescence, the pigeons were returned to the dovecote for group feeding.

### 2.4. Wireless Stimulation System

A block diagram of the wireless stimulation system illustrates the composition of the Bluetooth RF Serial Port Transfer Station (BRFSP Transfer Station), Micro Mobile Brain Square Wave Stimulator (MMB-SWS), and mobile phone applications (APP) (Figure 2a).

For the APPs, a variety of stimulation parameters can be adjusted, including stimulation channel, intensity, frequency, pulse width, and stimulation time. After setting, the BRFSP Transfer Station can be connected through Bluetooth and the parameters sent to the MMB-SWS in the form of an RF signal.

The BRFSP Transfer Station utilizes the CC2541 and CC1110 chips to facilitate the conversion between Bluetooth signals and RF signals, enabling wireless communication between APPs and MMB-SWS (Figure 2c).

The working voltage of the MMB-SWS is 3.7 V powered by a lithium battery, and the circuit is shown in the figure. Using the CC1110 wireless communication chip, once it receives the parameters, the digital signal is transformed into an analog voltage signal through the AD5310 chip. Subsequently, the signal is amplified by the AD820 and, finally, eight channels of stimulation output are achieved via the CD4051 multi-channel analog switch chip. The stimulation pulses generated are of a biphasic current nature. Within the stimulator, two REG710NA-5 and REG710NA-3-3 components serve as regulators to provide stable power supply voltage. The circuit generates a negative voltage using the TC7660 chip. The CC1110 wireless communication chip is also connected to two LED indicator lights, which illuminate when the stimulator is powered on or when stimulation pulses are being output (Figure 2c).

### 2.5. Electrical Stimulation Protocols

To ensure the efficacy of electrode implantation, we administered electrical stimulation during the surgical procedure while simultaneously implanting the electrodes. In the electrostimulation experiments conducted under light anesthesia, we employed the YC-2-S bipolar programmable stimulator (Chengdu Instrument Factory, Chengdu, China). The initial intensity was set to 2.5 V, with a pulse duration of 1.0 ms and a frequency of 80 Hz. When observing the pigeon’s responses to the stimulation, we implemented a step size of 200 μm when the pigeon exhibited no discernible reactions. However, when the pigeon displayed responses to the stimulation, we reduced the step size to 100 μm. The region capable of eliciting the maximum response was defined as the target area for electrode implantation. Subsequently, we gradually decreased the stimulation intensity until the minimum stimulation intensity did not fall below 0.5 V, in order to achieve movement patterns and intensities similar to the pigeon’s natural responses. Once the electrode implantation site was confirmed, we administered three stimulations, spaced 3 min apart, to ensure an adequate correlation between stimulation and response. Throughout the experiment, response behaviors induced by stimulation were simultaneously recorded using a digital camera (Nikon S210, Tokyo, Japan).

To investigate the quantitative impact of electrical stimulation parameters on controlling pigeon steering behavior, we conducted electrical stimulation on pigeons equipped with an electrode adapter using a wireless stimulation system while the pigeons were in a freely awake state. Using the surgical procedure’s electrical stimulation parameters as a reference, we maintained a fixed stimulation frequency (80 Hz), pulse width (1.0 ms), and stimulation duration (3 s). The initial stimulation intensity was set at 1.2 V, with increments of 0.2 V in intensity, up to a maximum of 4.0 V. Stimulation intensity was determined based on the behavioral responses induced by the stimulation. When observable behaviors such as takeoff or vigorous struggle were detected, the increase in stimulation intensity was halted. Each group of stimulation parameters was repeated at least three times for all subjects, with an interval of 3 min. The above experiment began one week after the implantation of the BCI. Initially, the experiments were conducted once a day. Subsequently, the frequency was adjusted to once every three days after three trials, and then to once every seven days after another three trials. Finally, the experiment was repeated three times to completion.

### 2.6. Animal Behavior and Motion Capture

**Figure 3:**
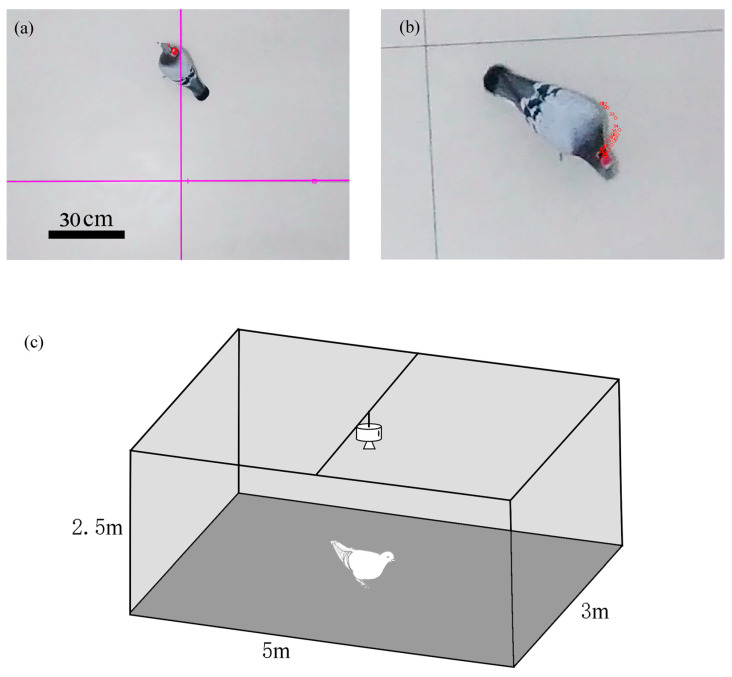
Motion Capture. (**a**) Dove motion capture interface. (**b**) Recognition of the target area and captured tracks. (**c**) Schematic diagram of pigeon motion capture area.

The wireless stimulation system and the pigeon head electrode adapter were assembled, and then they could move freely for 10 min in the animal behavior movement capture area (Figure 3c) (the animal behavior movement capture area is located in a closed room, with the size of about 3 m × 5 m × 2.5 m) to adapt to the surrounding environment. The behaviors of pigeons such as steering, walking, or short-range flying induced by electrical stimulation were recorded and evaluated. The movement trajectory, velocity, acceleration, and angular velocity of pigeon head during electrical stimulation were recorded by an action camera and captured by Tracker motion tracking software (ver. 6.1.5). The camera’s shooting angle was a top view, which could record the movement track of pigeons on the ground. Metric scale rulers were placed on the floor to be taken by the camera to calculate the real-world scale in the video clip (Figure 3a).

In the process of behavior analysis, the wireless stimulator fixed on the head of the pigeon was selected as the marker (binding the wireless stimulator with red tape). Then several key frames were marked manually, and the program estimated the position of the pigeon in other consecutive frames. Finally, the frame was manually corrected with the mark point offset (Figure 3b).

### 2.7. Data Analysis

To quantitatively analyze the impact of varying voltage intensities on the steering and locomotion behavior of pigeons, we computed and recorded four pivotal parameters: the total distance (S), the average speed (v), the mean angular velocity ($\bar{\omega }$), and the average turning radius (*R*) of the stimulus-induced motion. Provided with real-time coordinates of the pigeon during electrical stimulation-induced motion, the total distance covered during the stimulation-induced movement can be calculated using the following formula.

(1) $$S=\overset{N-1}{\underset{i=1}{\sum }}\sqrt{{\left(x_{i+1}-x_{i}\right)}^{2}+{\left(y_{i+1}-y_{i}\right)}^{2}}$$

In this context, ‘S’ represents the total distance, ‘N’ stands for the number of data points, and ‘(x_i_, y_i_)’ denotes the coordinates at each time point. Each term in the equation signifies the distance between two consecutive data points, calculated through the Euclidean distance. By dividing the distance by time (Δ*t*), the average speed (v) can be obtained. Utilizing the aforementioned data and the duration of pigeon movement, the average angular velocity during electrical stimulation-induced movement can be computed as follows, where Δ*t* represents the total time within this interval.

(2) $$\bar{\omega }=\frac{\sum_{i=1}^{N-1}arctan\left(\frac{y_{i+1}-y_{i}}{x_{i+1}-x_{i}}\right)}{\Delta t}$$

By fitting the discrete points along the trajectory of pigeon movement, an approximate elliptical function can be obtained. Calculating the curvature of the function yields the curvature (*C*). According to the following formula, the turning radius (*R*) can be determined.

(3) $$R=\frac{1}{C}$$

Furthermore, we quantified the response time (Tr) of pigeons to electrical stimulation-induced steering behavior, which represents the time delay from the onset of electrical stimulation to the significant change in a pigeon’s trajectory. By employing the aforementioned approach, we were able to precisely quantify the behavioral responses of pigeons following electrical stimulation, offering a systematic solution for the quantitative analysis of pigeon steering movements. All analytical procedures were conducted on the platform MATLAB 2015b (The MathWorks, Inc., Natick, MA, USA).

### 2.8. Histology

Pigeons in this study were euthanized after all experimental tests. And then transcarotid perfusion was initiated with 0.8% sodium chloride (NaCl), followed by 4% paraformaldehyde (PFA). After the blood had been successfully exchanged with PFA, the brain was removed from the skull and stored in a postfix solution (4% PFA with 20%/30% sucrose) at 4 °C until the brain tissue settled into the bottom of the container. Brains were cut in a coronal plane in 30 µm-thickness using a freezing microtome. Neutral red staining was performed on brain tissue sections to determine the actual coordinates of electrodes.

### 2.9. Statistical Analyses

The number of successful steering and corresponding response times for each participant were tallied and represented as the mean ± standard error. The normality of the data’s distribution and the homogeneity of total travel distance, average angular velocity, and mean turning radius induced by stimulation were assessed using the Shapiro–Wilk W test and Levene’s test. All analyses were conducted using IBM SPSS Statistics 26.0 (IBM SPSS, Inc., Chicago, IL, USA), with a significance level set at *p* < 0.05.

## 3. Results

### 3.1. Motor Behavior Induced by Micro-Electrical Stimulation in Light Anesthesia State

All pigeons were induced to perform motor behavior under light anesthesia through micro-electrical stimulation. Under low-intensity stimulation, pigeons exhibited behaviors such as wing flapping, unilateral leg extension, and body rotation. The frequencies of wing flapping, unilateral leg extension, and body rotation were 28.57%, 28.57%, and 85.71%, respectively. As the stimulation intensity increased within a reasonable range, the pigeons’ response strength also increased accordingly. In a state of light anesthesia, the average threshold that induced motor behavior in pigeons was 2.39 ± 0.40 V (N = 24, number of electrode tracks).

### 3.2. Parameters in Free-Flight Tests

#### 3.2.1. Response Time and Success Rate of Steering Control

**Figure 4:**
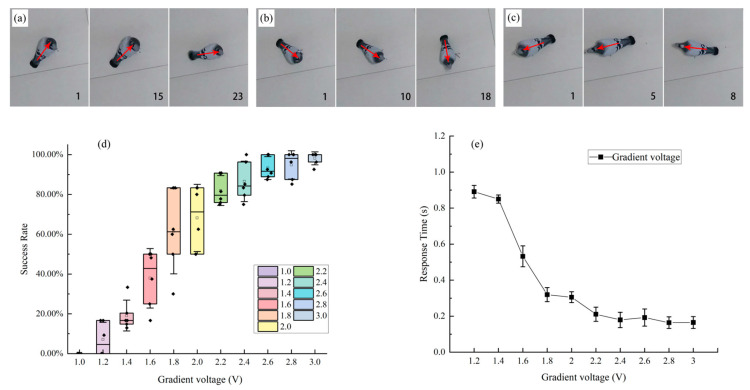
Starting and success rate of pigeon robot movement. (**a**) Pigeon response delay under 1.4 V stimulation (The red arrow originates from the tail and points towards the head of the pigeon, indicating the body orientation of the pigeon in the current frame. Select the start of the stimulus as the first frame, frequency 30 frames per second). (**b**) Pigeon response delay under 1.8 V stimulation. (**c**) Pigeon response delay under 2.2 V stimulation. (**d**) The success rate of steering controlled by gradient voltage stimulation parameters. (**e**) Motion response time under gradient voltage stimulation.

Figure 4d demonstrates the induction success rate of steering motion in the pigeon robot under gradient voltage stimulation. Figure 4e shows the response time of steering motion in the pigeon robot under gradient voltage stimulation (*p* < 0.05). The minimum voltage at which the pigeon robot can generate responsive movements is 1.2 V (with a total success rate of 4.17%), and noticeable behavioral movements begin to appear at a widely seen 1.8 V voltage stimulation (with a total success rate of 58.33%). Generally, as the voltage increases, the response delay of pigeons to electrical stimuli decreases (Figure 4a–c). When the voltage intensity exceeds 1.8 V, there is no longer a significant change in response delay (*p* > 0.05). Under reasonable stimulation intensities (1.8–3.0 V), the average delay time for electrical stimulation-induced pigeon movements is 220 ms.

#### 3.2.2. Effects of Different Electrical Stimulation Parameters on Pigeon Steering Behavior

**Figure 5:**
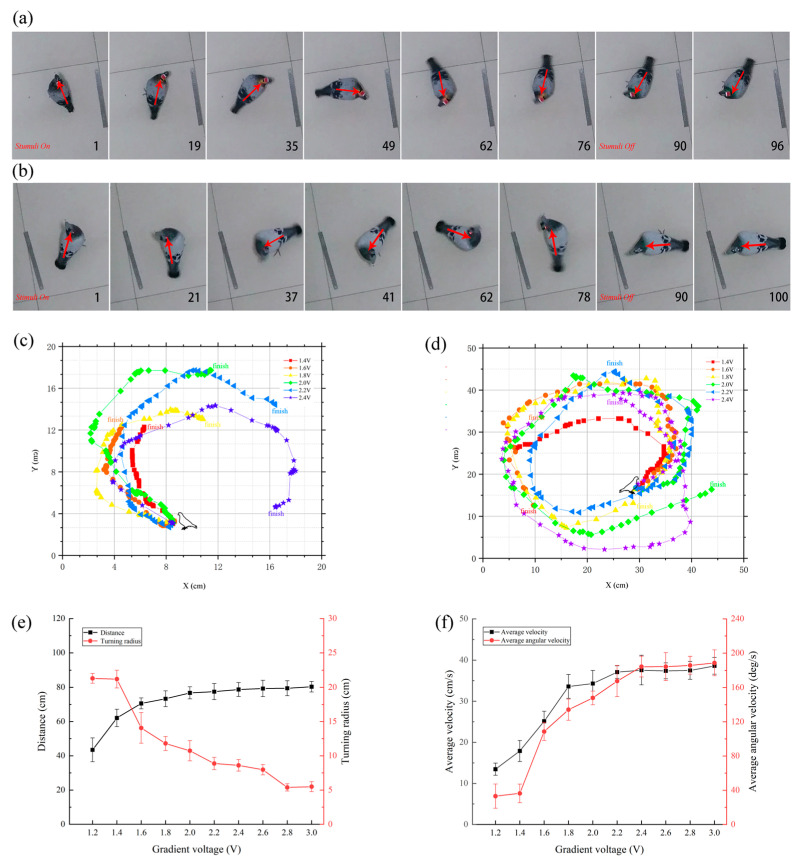
Stimulation of FRM-induced movement during freely moving test. Pictures from left to right in each line indicate successive time during the freely moving test. Numbers on each frame indicate the serial number of the video frame (frequency 30 frames per second). The red arrow originates from the tail and points towards the head of the pigeon, indicating the body orientation of the pigeon in the current frame. (**a**) Right-turning (the stimulus parameter is 2.2 V). (**b**) Left-turning (the stimulus parameter is 2.2 V). (**c**) Clockwise rotation path capture image. (**d**) Counterclockwise rotation path capture image. (**e**) Comparison of total distance and average turning radius under gradient voltage stimulation. (**f**) Comparison of average velocity and average angular velocity under gradient voltage stimulation.

As shown in Figure 5 and Table 1, under different voltage intensities, pigeons were successfully induced to exhibit steering behavior through electrical stimulation of the FRM (Figure 5c,d). In the free and awake state, pigeons exhibited stable and repeatable steering behavior upon receiving stimulation signals from the wireless stimulation system. Under gradient voltage stimulation, within the same stimulation duration, there was no significant difference (*p* > 0.05) observed in the total distance traveled and the average speed of pigeon motion as the voltage intensity increased (Figure 5e,f). However, the angular velocity and turning radius of the robotic pigeon varied with different stimulation parameters (Figure 5e,f). With an increase in stimulation voltage intensity, the angular velocity of the robotic pigeon gradually increased, the turning curvature decreased, and the turning radius enlarged. Considering that excessively high voltages or prolonged stimulation times may cause serious damage to brain tissue in behavioral control studies, the test voltage range was restricted to 1–3 V, the stimulation frequency was set at 80 Hz, and the duration of the stimulation was uniformly fixed at 3 s.

#### 3.2.3. Effectiveness of Post-Implantation Control

The induced behavioral actions were carried out 7 days post electrode implantation, considering the changes in brain tissue or the surrounding environment caused by prolonged electrode implantation. After a round of stimulation, the pigeons were released back to the dovecote for free feeding, and the electrical stimulation experiments were repeated one month after the completion of the electrode implantation period. In comparison with the initial experiment, there was no significant change (*p* > 0.05) in the response time of the pigeons to electrical stimulation, but the minimum voltage required to produce responsive movements changed from 1.2 V to 1.6 V. Under the same stimulation parameters, there was no significant change (*p* > 0.05) in the motion parameters. These results indicate that even after one month of electrode implantation, electrical stimulation can still consistently induce steering motion in the pigeon robot, and the quantitative control of motion behavior by stimulation parameters remains stable. Furthermore, the increase in the threshold voltage is attributable to a decrease in the sensitivity of the brain region to electrical stimulation, which is likely a result of the prolonged implantation time of the electrodes and the increased number of stimulation events.

## 4. Discussion

This study demonstrates that in the pigeon robot, gradient voltage stimulation of the FRM region can elicit different turning behaviors (Figure 5). Generally, when controlling the turning behavior of a pigeon robot through a wireless stimulation system, the turning behavior occurs immediately after a brief delay of milliseconds, and the turning speed and angle are influenced by the stimulation voltage intensity. Within the normal range, a higher stimulation voltage results in faster turning speeds and larger turning angles for the pigeon robot (Figure 5e,f). In earlier studies, the control of the pigeon robot was only qualitative, without achieving quantitative control. Cai et al. [24] found as early as 2015 that applying electrical stimulation to the FRM or vestibular dorsal lateral nucleus (VeDL) region of pigeons with fixed heads under light anesthesia would trigger body tilting to the left or right. Quantifying the effects of stimulation parameters on motion behavior facilitates engineers in developing pigeon robots and bionic pigeon robots more easily, providing solutions for encoding complex movements of pigeon robots and enabling the precise manipulation of pigeon robot flight actions. Therefore, this study complements experiments involving electrical stimulation applied to freely awake pigeons, triggering controlled left and right turns in pigeons, proving that stimulation voltage intensity is a key factor in activating neurons to generate action potentials, thus achieving quantitative control of the steering behavior of pigeon robots.

### 4.1. Relationship between FRM and Motion Behavior

The above study demonstrates the significant impact of electrical stimulation parameters on steering behavior in pigeons induced by FRM stimulation. Current research on avian brain structures includes the hippocampus, PoA, nidopallium caudolaterale (NCL), basal ganglia, cerebellum, and midbrain, which have been more finely divided into subregions [22,24,31,32,33]. The FRM is located in the medial part of the mesencephalic reticular formation (MRF). The avian MRF primarily projects to the tectum, cervical spinal cord, and medial reticular formation, and descending pathways from the forebrain to the midbrain are believed to be associated with motor functions [34]. Our findings are consistent with previous research, indicating a close relationship between FRM and pigeon steering motion. Quantitative analyses of the effects of gradient stimulation voltage intensity on the motion distance, average angular velocity, and turning radius of the pigeon robot revealed that as the stimulation voltage gradient increased, the motion distance and steering angle of the pigeon robot were positively correlated with the stimulation parameters. However, when the stimulation voltage intensity reached a certain threshold (>1.8 V), even with further increases in voltage, there was no significant difference in the steering speed of the pigeon (*p* > 0.05), while the average angular velocity increased with the stimulation voltage intensity (*p* < 0.01). As a compensatory effect, the turning radius of the pigeon decreased, and the curvature increased (*p* < 0.01). From this, we infer that the FRM region is mainly responsible for regulating body rotation, with relatively minor impact on motion speed.

### 4.2. Impact of Gradient Voltage Variation on Electrical Stimulation Effects

Previous studies have indicated that the excitation of neuronal action potentials requires an adequate amount of current, which is proportional to the square of the distance between the neuron and the electrode tip, and the degree of neuronal activation is related to the applied effective charge [35,36]. Increasing the voltage intensity can activate a larger range of neurons, which is the fundamental reason why the response delay to electrical stimulation under low voltage conditions is greater than under high voltage conditions. When the intensity of the voltage exceeds 1.8 V, the response-delay of the pigeon robot no longer changes significantly. This observed phenomenon can be attributed to a threshold effect. When using electrical stimulation to activate specific brain regions, increasing the voltage does augment the number of activated neurons, potentially enhancing certain types of neural responses. However, once the voltage reaches a saturation point, nearly all target neurons are engaged. Beyond this point, further increases in voltage do not significantly enhance the response intensity or reduce the reaction latency. Additionally, in animal experiments, stimuli of different intensities within the same brain area may elicit different behavioral responses. Many animal experiments have shown that stimuli of different intensities can induce different behavioral responses. For example, Cabelguen et al. found that in the same area with different stimulus intensities, different movement patterns could be induced. During low-intensity stimulation, only limb movements were induced, while increasing the stimulus intensity could achieve alternating swimming movements [37]. This may be attributed to the number of neurons related to activated behaviors. When the stimulus intensity equals or slightly exceeds the threshold, only a small portion of neurons are excited, resulting in only a small number of motor units being activated. As the stimulus intensity increases, the number of excited motor-related neurons increases, thereby activating more motor units and producing a stronger response. In this study, we set the stimulus intensity at 1 V, with an increment of 0.2 V for each stimulation. During the trajectory analysis, if the marker moves more than twice within the stimulus period and the distance of movement exceeds 5 cm, it is considered a valid response. Among all experimental animals (N = 6, number of electrode tracks = 24), the number of electrode tracks inducing behavioral actions at 1.2 V was 2, and the voltage strength generally inducing pigeon turns was 2.0 V. When the voltage was less than 2.0 V, there was a significant delay in pigeon turning movements (*p* < 0.05), and the speed and angular velocity were slower. However, when the stimulation voltage exceeded 2.6 V, it could potentially alter the pigeon’s movement pattern, changing the stepping and turning behavior on the ground to circling in flight, while maintaining the same turning direction. In our experimental group of six pigeons, three exhibited changes in their motor behavior. Furthermore, with the increase in voltage intensity, the response time of the pigeon robot to electrical stimuli gradually decreased, until it reached the threshold voltage intensity (2.2 V), after which, the response time stabilized at around 220 ms. Overall, the changes in turning movements were closely related to the stimulus intensity. Based on these results, the appropriate stimulus intensity can be chosen to control the pigeon’s movement according to individual needs.

### 4.3. Motion Analysis

Due to the difficulty in directly observing animals during movement, researchers often analyze the characteristics of animal motion paths. The current scheme for the indoor trajectory analysis of pigeon movement is immature and does not effectively quantify various indicators of pigeon motion. Real-time trajectory and velocity are common quantitative assessment indicators in animal motion trajectory analysis [38]. In addition, when it comes to behaviors such as turning, turning angle and turning radius are also important quantitative indicators [39]. Animal motion trajectories are often defined as a set of time-specific discrete positions, usually confined to a two-dimensional or three-dimensional system. As animal motion is continuous, the trajectory is obtained by repeatedly sampling the animal’s position over time [40]. The Tracker software can be used to statistically analyze pigeon motion trajectories, and its windowed operation is more user-friendly than programming methods [41]. In this paper, we quantified the motion trajectories of pigeon turning behavior in a two-dimensional coordinate system. In future studies, animal motion trajectories can be captured using dual-camera setups, establishing a Cartesian coordinate system to achieve a quantitative analysis of pigeon flight, circling, and other motion behaviors in three-dimensional space.

## 5. Conclusions

In this study, we quantitatively analyzed the changes in various parameters of pigeon steering behavior induced by adjusting voltage intensity gradients. This analysis provides valuable reference for accurately encoding actions for pigeon robots. The alteration of voltage intensity (1.2–3.0 V) significantly affects the angular velocity and turning radius during pigeon movement. Beyond the threshold voltage, changes in voltage intensity do not significantly impact the distance and average speed of pigeon movement. Therefore, pigeon robots based on electrical stimulation of the FRM region can accomplish prescribed steering actions under different voltage intensities. Additionally, the motion analysis process in this study also provides references for interpreting pigeon motion indicators. In the future, we aim to further quantify the effects of different stimulus parameters from other brain regions on pigeon locomotion and gradually explore solutions for the complex coding of multi-brain regions to optimize control schemes for pigeon robots.

## Figures and Tables

**Table 1:** Results of the pigeon robot steering controlled with the varied parameters of voltage intensity.

**Stimulus Intensity** **(V)**	**Distance** **(cm)**	**Velocity** **(cm/s)**	**Angular Velocity** **(deg/s)**	**Turning Radius** **(cm)**	**Response Time** **(s)**
1.2	43.45 ± 6.95	13.45 ± 1.48	33.11 ± 14.16	21.30 ± 0.70	0.89 ± 0.04
1.4	62.1 ± 5.10	17.89 ± 2.55	36.34 ± 10.74	21.20 ± 1.30	0.85 ± 0.02
1.6	70.57 ± 3.19	25.13 ± 2.45	108.55 ± 10.45	14.07 ± 2.24	0.53 ± 0.06
1.8	73.30 ± 4.63	33.60 ± 2.90	134.13 ± 12.36	11.80 ± 1.02	0.32 ± 0.04
2.0	76.75 ± 3.52	34.31 ± 3.19	147.82 ± 7.97	10.73 ± 1.48	0.31 ± 0.03
2.2	77.45 ± 4.75	37.10 ± 1.53	167.57 ± 18.30	8.88 ± 0.90	0.21 ± 0.04
2.4	78.70 ± 4.05	37.55 ± 3.57	184.35 ± 12.03	8.60 ± 0.85	0.18 ± 0.04
2.6	79.30 ± 4.78	37.39 ± 1.94	184.42 ± 16.22	7.97 ± 0.75	0.19 ± 0.05
2.8	79.42 ± 4.37	37.50 ± 2.16	185.86 ± 10.28	5.38 ± 0.53	0.16 ± 0.03
3.0	80.33 ± 3.07	38.61 ± 2.02	188.70 ± 15.01	5.49 ± 0.72	0.16 ± 0.03

## Data Availability

The data that support the findings of this study are available on request from the corresponding author, Lei Cai, upon reasonable request.
